# It's only natural: Plant respiration in unmanaged systems

**DOI:** 10.1093/plphys/kiad167

**Published:** 2023-03-21

**Authors:** Stephanie C Schmiege, Mary Heskel, Yuzhen Fan, Danielle A Way

**Affiliations:** Plant Resilience Institute, Michigan State University, East Lansing, MI, 48824, USA; Department of Biology, Western University, N6A 3K7, London, ON, Canada; Department of Biology, Macalester College, Saint Paul, MN, USA 55105; Research School of Biology, The Australian National University, Acton, ACT, Australia; Department of Biology, Western University, N6A 3K7, London, ON, Canada; Research School of Biology, The Australian National University, Acton, ACT, Australia; Environmental & Climate Sciences Department, Brookhaven National Laboratory, Upton, NY, USA; Nicholas School of the Environment, Duke University, Durham, NC, USA

## Abstract

Respiration plays a key role in the terrestrial carbon cycle and is a fundamental metabolic process in all plant tissues and cells. We review respiration from the perspective of plants that grow in their natural habitat and how it is influenced by wide-ranging elements at different scales, from metabolic substrate availability to shifts in climate. Decades of field-based measurements have honed our understanding of the biological and environmental controls on leaf, root, stem, and whole-organism respiration. Despite this effort, there remain gaps in our knowledge within and across species and ecosystems, especially in more challenging-to-measure tissues like roots. Recent databases of respiration rates and associated leaf traits from species representing diverse biomes, plant functional types, and regional climates have allowed for a wider-lens view at modeling this important CO_2_ flux. We also re-analyze published data sets to show that maximum leaf respiration rates (*R*_max_) in species from around the globe are related both to leaf economic traits and environmental variables (precipitation and air temperature), but that root respiration does not follow the same latitudinal trends previously published for leaf data. We encourage the ecophysiological community to continue to expand their study of plant respiration in tissues that are difficult to measure and at the whole plant and ecosystem levels to address outstanding questions in the field.

## Teaser

Plant respiration varies in natural ecosystems—across leaves, stems, roots, and the whole organism—creating global patterns in respiration across species, ecosystems, and biomes.

## Introduction

Mitochondrial respiration in plants consumes about half of the carbon fixed by photosynthesis annually (Chapin et al. 2012), producing energy to fuel metabolism and growth, releasing carbon skeletons for biochemical processes, and balancing cellular redox status (O’Leary et al. 2019). Respiration is thus a critical process for plant productivity and survival. The balance between photosynthesis and respiration in plants also helps determine atmospheric global CO_2_ concentrations (Amthor 1995; Ciais et al. 2013). Understanding respiration in natural ecosystems is therefore not only important for improving our ability to model and manage non-agricultural systems, but also for predicting the trajectory of future climate change (Dusenge et al. 2019).

Respiration can be defined in multiple ways (O’Leary et al. 2019). Researchers in mitochondrial biology usually focus on the metabolic pathways and regulation of glycolysis, the tricarboxylic acid (TCA) cycle, and the mitochondrial electron transport chain (O’Leary and Plaxton 2016). However, this level of work is rarely done on vegetation in the field. Instead, as noted by Ryan (1991), ecologists and (eco)physiologists tend to define respiration in terms of CO_2_ release rather than through a biochemical lens, where O_2_ uptake would be more relevant given its coupling with respiratory ATP production (Plaxton and Podestá 2006). We will therefore focus on CO_2_ efflux measurements, as these are the most common data for plants from natural ecosystems (but see Box 1).

Box 1. **The respiratory quotient in natural vegetation**

Both O_2_ and CO_2_ fluxes are sometimes assessed on the same tissue to measure the respiratory quotient (RQ, the ratio of CO_2_ efflux to O_2_ uptake). The RQ provides insight into the types of substrates fueling respiration: compounds such as carbohydrates yield a respiratory quotient of unity, whereas a more reduced substrate generates an RQ below 1, with RQ values of 0.8 to 0.9 for proteins and as low as 0.7 for lipids (Tcherkez et al. 2003; Araújo et al. 2011). Work on both Scots pine (*Pinus sylvestris*) seedlings and French bean (*Phaseolus vulgaris*) showed that the RQ of leaves exposed to normal irradiance during the day was near unity, but declined as plants were maintained in darkness over multiple days, indicating a gradual switch towards using lipids for respiration under carbohydrate starvation (Tcherkez et al. 2003; Fischer et al. 2015). Hanf et al. (2015) also found that seedlings of *P. sylvestris* and Norway spruce (*Picea abies*), co-occurring boreal trees, moved from burning only carbohydrates to using a mix of substrates for respiration during shading, but that only *P. sylvestris* (a lipid-storing species) did so under water stress, an ability which may contribute to the pine species’ greater drought tolerance in nature. Although there are relatively few studies that assess the RQ of vegetation, there are data on RQ from field-grown trees. Hilman et al. (2019) found that the measured RQ on stems from nine species was less than unity, with many values < 0.7. The authors concluded that the observations of low RQ values may be a result of ∼40% of the CO_2_ respired by the stem tissue not being released at the local stem surface. In a different study assessing leaves of 9 co-occurring tree species, the RQ was close to unity for species growing near the center or northern edge of their range, but greater than unity in species that typically grow north of the study site, implying that these cool-adapted species could not rely on carbohydrates alone for respiration (Patterson et al. 2018). The same study also found that the RQ in field-grown leaves increased 14% as measurement temperatures rose from 15 °C to 35 °C (Patterson et al. 2018), a pattern which differs from data collected in earlier lab-based studies (Tcherkez et al. 2003).

By focusing on naturally occurring vegetation, we (naturally…) raise the question of what we mean by natural ecosystems. Given the planetary scale of rising CO_2_ concentrations and other global change factors, even plants in remote regions are affected by anthropogenic influence. However, we will use the terms “naturally occurring” and “field-grown” interchangeably to indicate vegetation in its natural habitat that is unmanaged or lightly managed by humans. This excludes agricultural systems, forestry plantations, and plants grown in chambers and greenhouses, while encompassing vegetation that is grown in the field but might experience some low-level management or experimental manipulation. Although the metabolic process of respiration is similar in all plants, there are key differences between natural and managed systems that may affect respiration. For example, crops are often annual species bred to maximize yield, which can lead to different strategies of carbohydrate usage and allocation, compared to perennials and many naturally occurring annuals. Additionally, both crops and plants from lab settings are often grown with ample water and nutrients, conditions that are less common in nature; these growth conditions may alter respiratory physiology, such as the degree to which leaves are substrate limited (McCutchan and Monson 2001). We will therefore refer to lab studies, since much of our understanding of respiratory biochemistry and physiology comes from these studies, but aim to highlight whether these results have been confirmed in the field.

Unlike photosynthesis, respiration occurs both during daylight and in darkness. Accounting for respiration across a diel cycle is therefore important in natural systems, given the potential impact of daytime respiration (i.e. light respiration) for plant carbon budgets (Tcherkez et al. 2017). There is an entire literature on light respiration, with the general conclusion that light suppresses mitochondrial activities by ∼30%, although light respiration can be anywhere from 16% to 140% of dark respiration (Tissue et al. 2002; Crous et al. 2012; Weerasinghe et al. 2014; Kroner and Way 2016). However, there is considerable debate surrounding the appropriate techniques for measuring light respiration. We will thus concentrate on dark respiration in this review, but interested readers are directed to a series of recent papers on light respiration (Buckley et al. 2017; Farquhar and Busch 2017; Tcherkez et al. 2017; Keenan et al. 2019; Way et al. 2019; Gauthier et al. 2020; Xu et al. 2021).

**Figure 1.**
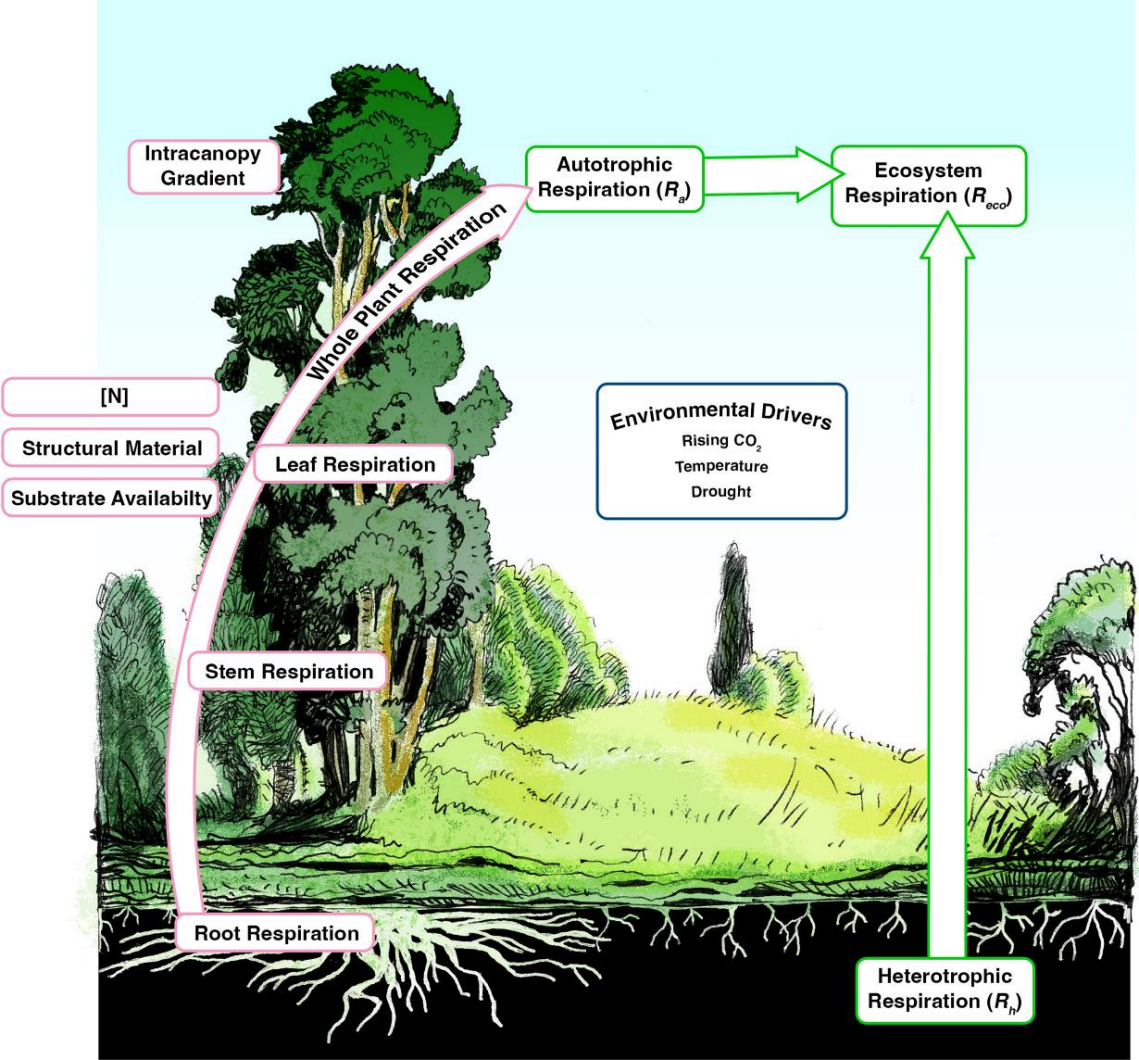
A conceptual figure of the topics covered in this update. Red outlined boxes on the left represent how tissue respiratory CO_2_ fluxes and the factors that modify these fluxes scale to whole-plant respiration. Green outlined boxes represent ecosystem respiratory CO_2_ fluxes, including the sum of whole-plant respiration from the ecosystem (autotrophic respiration) and respiration from soil microbes (heterotrophic respiration). The blue outlined box in the centre shows environmental drivers of plant dark respiration.

In this review, we discuss (i) the internal biological factors that are correlated with respiration in plants growing in natural systems; (ii) how respiration is measured across biological scales in the field; and (iii) major environmental and ecological causes of variability in respiration within and across plants (Fig. 1). Lastly, we will consider future directions for developing a better understanding of respiration in natural settings. This review brings together major findings on respiration in multiple tissue types and scales this up to discuss whole plant, ecosystem, and global patterns in respiration.

## Internal factors related to respiration rate

Respiration correlates with a number of internal factors in plants, including tissue nitrogen concentration, plant age and size, and substrate availability, as well as environmental drivers, including temperature, drought, and soil fertility (Piao et al. 2010; Fig. 1). Critically, the internal factors best linked to high respiration rates are indicators of plant vigor and growth potential, highlighting the close connection between respiration and plant productivity.

Tissue nitrogen concentration ([N]) is one of the strongest predictors of respiration (Wright et al. 2004; Atkin et al. 2015), accounting for approximately two-third of the variation in mass-based respiration rates across plant functional types (PFTs) (Reich et al. 2008). The relationship between [N] and mass-based respiration rate is ubiquitous because higher [N] indicates higher concentrations of N-rich proteins, enzymes, and compounds (such as chlorophyll), which fuel high metabolic rates and demands for adenosine triphosphate (ATP) and NADPH (Reich et al. 2006). The robust correlation between mass-based respiration and [N] underlies the broad use of [N] as a predictor for respiration in models (Ryan 1991; Thornley and Cannell 2000). The degree to which a given increase in [N] results in a rise in respiration rate is similar across leaves, stems, and roots; however, the respiration rate per unit N is lower in leaves than in other tissues (Reich et al. 2008). While the reason for this is unclear, it likely involves photosynthesis. Leaves can use ATP generated from photosynthesis, which may reduce their need for respiratory ATP (Cannell and Thornley 2000). Alternatively, the large investment of N into photosynthetic machinery may mean that less of the N in leaves is dedicated to respiration-oriented functions than is the case for stems and roots (Reich et al. 2008). The relationship between leaf [N] and mass-based respiration rates is also tightly linked to the leaf mass per area, as increases in structural carbon dilute tissue [N] and mass-based metabolic rates, relationships explained globally by the leaf economic spectrum (Wright et al. 2004).

One example of how leaf [N] and protein composition may alter respiration is given in C_4_ plants. As a result of their CO_2_-concentrating mechanism, C_4_ leaves have reduced photorespiration and increased photosynthesis, accompanied by ∼16% lower Rubisco concentrations than C_3_ leaves (Sage and Pearcy 1987; Ghannoum et al. 2005; Ghannoum et al. 2011; Evans and Clarke 2019). As Rubisco is the most abundant protein in leaves and requires respiratory ATP and carbon skeletons to turnover (Atkin et al. 2000b), low Rubisco concentrations may reduce respiratory demands in C_4_ leaves (Fan et al. 2022a). However, C_4_ leaves also exhibit higher concentrations of soluble proteins (to run the C_4_ cycle) and thylakoid proteins (Ghannoum et al. 2005; Ghannoum et al. 2011) than C_3_ leaves. The low Rubisco amounts in C_4_ leaves likely reduce respiratory demands, but these demands may be increased by turnover of these other proteins that have shorter half-lives than Rubisco (Simpson et al. 1981). Together, these trends may lead to similar leaf respiration rates in C_3_ and C_4_ plants (Byrd et al. 1992).

Respiration usually correlates with attributes related to the age or size of the tissue being measured, a trend associated with larger investments in carbon-rich (but metabolically inactive) structural material in long-lived tissues (Rodríguez-Calcerrada et al. 2012). Respiration also varies with root size/age, with younger roots generally exhibiting higher CO_2_ efflux rates than their older counterparts (Pregitzer et al. 1998). Such variation in root respiration is related to differences in metabolic activity and is correlated with root [N] (Pregitzer et al. 1998; Ben-Noah and Friedman 2018).

Within a given plant tissue, respiration rates depend not only on the biochemical and structural traits, but also on substrate availability (Cannell and Thornley 2000; Atkin and Tjoelker 2003; Fischer et al. 2015). Engaging photosynthesis or providing exogenous carbohydrates immediately before measuring respiration enhances leaf O_2_ uptake rates under lab conditions (Azcón-Bieto et al. 1983b; O’Leary et al. 2017). Similar results are seen in crops grown under elevated CO_2_ in the field where photosynthesis and carbohydrate concentrations are high (Leakey et al. 2009; Li et al. 2013). However, it is unclear whether the same phenomenon plays out in plants grown under less artificial conditions or in noncrop species. McCutchan and Monson (2001) found no relationship between nocturnal carbohydrate reserves and respiration in two perennial alpine plants and concluded that this uncoupling may facilitate carbon allocation to belowground storage tissues. Similarly, nighttime respiration was enhanced by both exogenous carbon and high daytime irradiance in spinach (*Spinacia oleracea*) (a high-light-demanding annual crop), evidence for substrate limitation, but unaffected by the addition of an uncoupler (used to remove adenylate restrictions; Lambers et al. 1998), indicating that respiration was substrate limited (Noguchi et al. 1996; Noguchi and Terashima 1997). However, the opposite was true for giant taro (*Alocasia macrorrhiza*), highlighting that respiration was more controlled by energy demand in this shade-tolerant perennial species (Noguchi et al. 1996; Noguchi and Terashima 1997). These papers raise the question of how substrate availability and product demand limitations interplay in field-grown plants. Overall, it is likely that both supply and demand limitations co-occur in natural settings (O’Leary et al. 2019). Indeed, under an optimality lens, plants should operate near a co-limited state of supply and demand limitations that would prevent overinvestment in respiratory proteins.

## How is plant respiration measured in natural systems?

Plant respiration in naturally occurring vegetation is commonly measured with gas exchange as either CO_2_ efflux or O_2_ uptake in the dark (Hunt 2003) (see Box 1), although both approaches capture signals from other biochemical processes, such as carboxylation via PEP carboxylase (O’Leary et al. 2019). Measurements of CO_2_ flux are commonly used in the field due to the difficulties in measuring small changes in O_2_ concentration against a background of 21% O_2_ outside of the lab (Helm et al. 2021). In contrast, O_2_ electrodes are commonly used on isolated mitochondria or detached tissue in lab settings (Loveys et al. 2003; Jacoby et al. 2015). Recently, a fluorometric oxygen sensor method has been pioneered for assessing leaf respiration rates (Sew et al. 2013; Scafaro et al. 2017), a technique that can be used in a high-throughput fashion to measure respiration from many plants simultaneously (e.g. O’Leary et al. 2017).

### Respiration of plant tissues in situ in the field

#### Leaves

While gas exchange measurements are easier to make on leaves than on stems or roots, leaves present their own challenges for assessing respiration. Leaf respiration is commonly assessed by clamping a gas-exchange cuvette onto leaf tissue and measuring the steady state CO_2_ efflux in the dark. Critically, the ability of leaves to photosynthesize means that the recent irradiance history of the leaf, and thus the local pool size of carbohydrates, can influence respiration. Leaf respiration declines as the duration of dark exposure increases, up to 20 to 30 min in the dark, when the respiration rate stabilizes (Azcón-Bieto et al. 1983b; Atkin et al. 1998). This means that leaves must be suitably “dark adapted” before respiration can be measured, which in the field often involves covering leaves in foil or dark cloth (Atkin et al. 2000a). Neglecting the dark-adaption period can lead to a post-illumination CO_2_ burst and light-enhanced dark-respiration, in which respiration is stimulated by the photorespiratory glycine shuttle and photosynthate supply, respectively (Atkin et al. 2000b). This phenomenon has been captured at the leaf-level (Atkin et al. 1998), as well as at the ecosystem scale (Barbour et al. 2007). The majority of leaf respiration data from field-grown plants is taken from dark-adapted leaves measured during daylight hours. However, measuring respiration at night (e.g. Aspinwall et al. 2016) ensures that only nocturnal biochemical processes occur. Indeed, recent work shows differences between respiration at the beginning and end of the night period, highlighting the need to measure nocturnal respiration, as even dark-adapted respiration values from the day may be misleading (Bruhn et al. 2022).

The largest data set on leaf respiration to date (GlobResp; Atkin et al. 2015) includes 899 species across 100 sites, but comparisons across this type of data set rely on using standardized leaf material. Fully expanded leaves are preferred because respiration declines as leaves expand and mature, both in the lab (Azcón-Bieto et al. 1983a; Priault et al. 2007) and the field (Collier and Thibodeau 1995; Shirke 2001; Xu and Baldocchi 2003; Kosugi and Matsuo 2006; Rodríguez-Calcerrada et al. 2012). Over the growing season, respiration in mature leaves tends to be stable until senescence begins, at which point respiration declines further, though CO_2_ efflux rates can spike at the onset of senescence (Collier and Thibodeau 1995; Crous et al. 2011; Heskel et al. 2014a; Heskel and Tang 2018). The light environment that a leaf matures in also influences the relative investment in structural material and metabolically active tissue, which affects respiration (sun vs. shade leaves; Lambers et al. 1998). It is therefore important to remember that published leaf respiration rates are not necessarily reflective of an average leaf.

#### Stems

Stem respiration is mainly measured in trees, as stems make up an increasingly large fraction of total biomass in woody species as plants ages (Poorter et al. 2012). Stem respiration can be estimated by attaching gas-exchange cuvettes to the tree stem. However, stem CO_2_ efflux is not a direct measure of stem respiration, as a variety of processes reduce CO_2_ release at the stem surface (Teskey et al. 2008; Hölttä and Kolari 2009; Angert et al. 2012). First, CO_2_ released from a section of stem tissue can diffuse into the xylem and move vertically via transpiration; CO_2_ from root and soil respiration can also diffuse into the xylem in the rhizosphere and move up into the measured stem segment (Teskey et al. 2008). Respired CO_2_ can diffuse axially within stem tissue, rather than immediately exiting at the local stem surface (De Roo et al. 2019). Additionally, stem-respired CO_2_ can be fixed by photosynthesis in the bark and subsurface tissues of young stems (Pfanz and Aschan 2001; De Roo et al. 2020) or via carboxylating enzymes such as PEP carboxylase (Hilman et al. 2019). Estimating stem respiration rates therefore requires information on not only stem CO_2_ efflux, but also sap flux rates to estimate CO_2_ transport rates (McGuire and Teskey 2004; Teskey and Mcguire 2007), and other variables such as stem temperature, combined with modeling to account for CO_2_ movement and losses via these processes (e.g. Salomón et al. 2022). To our knowledge, there is not yet a global analysis of stem respiration, but growing interest in this area should allow for broad data syntheses in the near future.

#### Roots

Root respiration in field-grown plants can be measured directly, whereby roots are carefully dug up and cleaned of soil before measuring gas exchange (Burton et al. 1998; Pregitzer et al. 1998). While this method is straightforward, some physical damage to the roots (especially fine roots) is inevitable, and change in the roots’ environment (e.g. humidity, light, and microbial interactions) will alter respiration in ways that are difficult to prevent. Root respiration can also be estimated indirectly by excluding autotrophic CO_2_ losses from soil, using root exclusion or tree girdling. In the root exclusion approach, roots are severed within the root exclusion plot, by trenching or installing root exclusion collars. Once the severed roots have decayed, paired measurements are made on root exclusion plots (to estimate heterotrophic respiration, *R*_h_) and nearby plots with intact roots (which measure both autotrophic respiration (*R*_a_) and *R*_h_). In the girdling method, bark is removed from trees, preventing carbon from being transported to the roots via the phloem; paired soil respiration measurements in girdled and ungirdled plots then allow for root respiration to be estimated (Högberg et al. 2001; Högberg et al. 2009). Root respiration can also be assessed using isotopes by providing a pulse of ^14^C and “chasing” it as it moves into biochemical pools and is eventually lost to the atmosphere or soil (Drake et al. 2019). However, partitioning the soil-respired CO_2_ into *R*_a_ and *R*_h_ is challenging and has limited the use of this approach in the field.

### Whole-plant respiration

Whole-plant respiration can be assessed by up-scaling tissue-specific respiration rates. But respiration can vary considerably between leaves across a canopy or stem tissues of various widths within an individual (Ryan et al. 1994), complicating this approach (Piao et al. 2010). The challenge in scaling up tissue-specific respiration can be seen in the wide variation in estimates of how whole-plant respiration is divided between tissues. Leaves can account for ∼50% of plant respiration (Atkin et al. 2007), although this changes with ontogeny (Armstrong et al. 2006). Stem respiration can be 5% to 40% of tree respiration (Salomón et al. 2022), depending on tree age and size. Roots can be responsible for 10% to 90% of soil CO_2_ efflux rates (Hanson et al. 2000), though a global analysis showed 30% to 50% of soil respiration came from roots (Bond-Lamberty et al. 2004). Despite these challenges, the up-scaling approach was used by Reich et al. (2006) to show that lab- and field-grown plants had a similar rise in respiration as they accumulated mass, but whole-plant respiration rates at a given size were smaller for naturally occurring plants than for lab-grown vegetation (Reich et al. 2006).

Whole-plant respiration can also be measured directly, though this is rare even in lab settings, given the need to measure root systems without contamination from soil processes. Estimates of whole-plant respiration using plants grown in inorganic media or hydroponic solutions have been made, as this circumvents most microbial respiration (Gifford 1995; Atkin et al. 2007; Wertin and Teskey 2008; Slot and Kitajima 2015a). In one of the few studies to directly assess whole-plant respiration in naturally occurring vegetation, Mori et al. (2010) found that the allometric exponent used to characterize size-dependent changes in respiration varied across plants spanning nine orders of magnitude in mass. Small plants are predominantly made of metabolically active leaf and root tissues, such that increases in plant mass produce a linear increase in respiration. However, larger plants increasingly require less metabolically active structural tissue for support and transport. The relatively low respiration rates of these structural components underlie the transition towards a three-fourth allometric exponent in the plant mass-respiration relationship as plant size grows (Mori et al. 2010).

### Plant respiration at the ecosystem level

Ecosystem-level CO_2_ fluxes from eddy covariance measure net ecosystem CO_2_ exchange (NEE), which is equivalent to ecosystem respiration (*R*_eco_) at night (when there is no gross primary production, GPP, i.e. ecosystem-level photosynthesis). In turn, *R*_eco_ is the sum of *R*_a_ from leaves, stems, and roots, plus CO_2_ release from *R*_h_, related to soil microbial activity (nighttime NEE = *R*_eco_ = *R*_a_ + *R*_h_; Fig. 1). Thus, at the ecosystem scale, plant respiration can be determined using eddy covariance (to measure NEE) combined with techniques such as girdling (Högberg et al. 2001) or root trenching (Järveoja et al. 2020) to isolate *R*_h_. Alternatively, *R*_a_ can be estimated from the difference between GPP and net primary production (NPP, estimated from carbon allocated to plant tissues). This approach relies on data from eddy covariance, with the uncertainties inherent in estimating GPP from NEE, and site-level accounting of leaves, stems, and roots, which introduces errors due to the inability to account for carbon allocated to nonstructural uses, such as root exudates (Piao et al. 2010). Lastly, *R*_a_ can be estimated with an up-scaling approach at the ecosystem scale, measuring leaf, stem, and root respiration with gas exchange and modeling CO_2_ efflux per unit ground area (Ryan et al. 1997). Remote sensing also offers exciting opportunities for estimating respiration at large spatial scales, though this approach is not yet well developed (Box 2).

Box 2. **Remote sensing of respiration**

The ability of remote sensing tools to estimate plant physiology has exploded in recent years. There are numerous remote sensing tools that can estimate plant CO_2_ uptake (as GPP), including solar-induced fluorescence (SIF) and vegetation indices such as the photochemical reflectance index (PRI) (Yoder and Waring 1994; Gamon et al. 2015; Gu et al. 2019; Magney et al. 2019). However, our ability to estimate plant CO_2_ efflux using similar tools is limited. Estimations of ecosystem respiration on large spatial scales often rely on the fact that temperature is a key determinant of respiration (Still et al. 2021). By combining remote sensing measurements of land surface temperatures with established temperature-respiration relationships, broad-scale patterns in ecosystem (or canopy) respiration can be obtained (Rahman et al. 2005).

Another approach for estimating leaf and canopy respiration with remote sensing is to use hyperspectral data. These data can then be correlated with data from the same vegetation on leaf [N] (as a proxy for respiration) or respiration rates captured via gas exchange. Given the strong relationship between respiration rate and leaf [N], remote sensing measurements of canopy [N] could be extrapolated to estimate canopy respiration on a wide spatial scale. In one of the few studies to attempt to directly link respiration with hyperspectral data, Coast et al. (2019) found that spectral data were better correlated with leaf [N] (r^2^ = 0.91) than with respiration rates (r^2^ = 0.5 to 0.63) in wheat (*Triticum aestivum*). To our knowledge, there are no studies using a hyperspectral approach to directly assess respiration in naturally occurring vegetation.

Globally, analyses of studies using eddy covariance and up-scaling found that annual forest *R*_a_ was strongly correlated with mean annual temperature (MAT), with a *Q*_10_ (the proportional change in respiration for a 10 °C increase in tissue temperature) of 1.8 to 2.9 (Piao et al. 2010), and that annual *R*_a_ increased with mean annual precipitation up to ∼2000 mm yr^−1^ (Morgan et al. 2021). Additionally, while annual *R*_a_ estimates from up-scaling increased with increasing stand biomass, leaf area index, and forest height, this was not the case with annual *R*_a_ calculated from the other methods, indicating that these significant relationships were spuriously caused by the circularity introduced by scaling local measurements with stand-level characteristics (Piao et al. 2010).

## Patterns of respiration within a canopy

Capturing respiratory variation throughout plant canopies is important for scaling respiration from leaves to the whole plant (Fig. 1). A 44% decrease in respiration with canopy depth has been found in temperate (Mitchell et al. 1999; Griffin et al. 2001; Griffin et al. 2002; Tissue et al. 2002; Turnbull et al. 2003; Whitehead et al. 2004a; Araki et al. 2017; Griffin et al. 2022; Schmiege et al. 2022), tropical (Cavaleri et al. 2008; Weerasinghe et al. 2014; Carter et al. 2021), and tropical montane cloud forest species (Van De Weg et al. 2012). However, in one of the only boreal species examined, white spruce (*Picea glauca*), no intracanopy differences in respiration were found at the northern treeline, while respiration decreased with canopy depth at the southern range extreme, likely due to changes in crown structure and local light environment (Griffin et al. 2022; Schmiege et al. 2022). Weak or no relationship of respiration with canopy position has been found in some conifer studies (Bond et al. 1999), while others showed decreases in respiration with canopy depth (Brooks et al. 1991; Araki et al. 2017), highlighting the need for further work in these species.

Intracanopy changes in respiration are correlated with biochemical and environmental gradients in temperature, humidity, wind, and light (Whitehead et al. 2004b). Greater irradiance correlates with higher respiratory activity, and upper-canopy leaves also have high carbohydrate concentrations (Griffin et al. 2001; Turnbull et al. 2003; Whitehead et al. 2004a), [N] (Reich et al. 1998), leaf mass per area, and mitochondria per leaf area (Reich et al. 1998; Tissue et al. 2002), supporting the idea that respiration is highest where metabolic activity is highest.

## Short-term temperature effects and thermal acclimation of respiration

### Short-term temperature effects on respiration

Of the environmental factors that influence plant respiration rates (Fig. 1), temperature is one of the most important. Respiration increases with a short-term rise in temperature (up to a temperature of >50 to 55 °C) (Heskel et al. 2014b; Heskel et al. 2016), largely because the maintenance costs of processes such as protein turnover increase under warmer conditions (Ryan 1991). Several methods have been employed to examine temperature responses of respiration, including the *Q*_10_, the activation energy (*E*_o_, determined by fitting a modified Arrhenius function to the respiratory temperature response), and a second-order log-normalized polynomial model (Heskel et al. 2016). In leaves, a *Q*_10_ of 2 is usually seen when respiration is measured near room temperature, although the *Q*_10_ declines at higher measurement temperatures (Tjoelker et al. 2001b; Atkin and Tjoelker 2003). In roots, the *Q*_10_ may be somewhat higher (2.4 to 3.1 in tree roots), though these high values may be due to measuring at low temperatures (6 to 24 °C; Burton et al. 2002). Because respiration in natural vegetation is so closely tied to tissue temperature, respiration is often modeled using a basal respiration rate (respiration measured at 25 °C) and then scaled with a temperature function (e.g. Heskel et al. 2016; Liang et al. 2018) or a *Q*_10_ (e.g. Wythers et al. 2013).

Temperature interacts with other drivers of respiration in natural ecosystems. An example of this is seen in boreal roots (Järveoja et al. 2020), where root respiration showed bimodal daily peaks. The first peak occurred near midday, when irradiance and air temperatures were near their peak, likely due to high substrate supply of photosynthates, whereas the second occurred late in the day, and was likely related to direct temperature effects from soil warming (Järveoja et al. 2020). Additionally, thetwo main conceptual subcategories of respiration, maintenance respiration and growth respiration (Thornley 1970; Amthor 1984; Cannell and Thornley 2000), can respond differently to short-term changes in temperature (Slot and Kitajima 2015b), since the metabolic processes supported by maintenance respiration are more temperature dependent than is growth on short timescales (Arcus et al. 2016).

### Thermal acclimation of respiration

The instantaneous temperature response of respiration described above varies depending on the plant's thermal history. Plants exposed to warming usually show a reduction in leaf basal respiration compared to control plants (Atkin and Tjoelker 2003; Slot and Kitajima 2015b; Zhu et al. 2021). The degree of thermal acclimation is similar between different biomes and PFTs, and in controlled environment studies and field-grown vegetation (Slot and Kitajima 2015b; Zhu et al. 2021) implying that a single function can be used in global models (Vanderwel et al. 2015; Slot and Kitajima 2015b). However, there is some variation in how much thermal acclimation occurs. Across 19 alpine species, thermal acclimation ranged from complete (i.e. respiration measured at the growth temperature was comparable between plants exposed to 10 °C and 20 °C), to almost nil (Larigauderie and Körner 1995). Within evergreen woody species, gymnosperms show a greater degree of thermal acclimation than broad-leaved species (Crous et al. 2022). Additionally, leaves that develop under warmer temperatures show greater acclimation than leaves that developed before warming occurs (Slot and Kitajima 2015b). The degree of thermal acclimation is sometimes correlated with leaf [N]. For example, basal respiration increased in trees exposed to warming compared to control trees, a result that correlated with higher leaf [N] in the warm-grown plants (Crous et al. 2017). Lastly, canopy position can also affect the thermal sensitivity of respiration, though the response is inconsistent: Griffin et al. (2002) found that the *Q*_10_ and *E*_o_ were smaller in upper-canopy leaves than lower canopy foliage, Turnbull et al. (2003) found the opposite, and many studies see no change in respiratory temperature sensitivity with canopy height (Xu and Griffin 2006; Cavaleri et al. 2008; Araki et al. 2017; Carter et al. 2021; Griffin et al. 2022).

At larger scales, little is known about how plant respiration at the ecosystem level (*R*_a_) adjusts to long-term changes in temperature. Globally, *R*_eco_ is more temperature sensitive in cold regions than in warm sites and, similar to leaf measurements, the *Q*_10_ of *R*_eco_ declines from the Arctic to the tropics (Johnston et al. 2021). The temperature sensitivity of *R*_eco_ also changes over the season as the temperature (and other environmental factors) changes (Reichstein et al. 2005). While it is unclear whether these results hold for *R*_a_, this similarity implies that there may be consistent temperature responses in respiration across biological scales.

## Variation in the temperature response of respiration: *t*  _max_ and *r*  _max_

Climate change makes it increasingly important to understand global patterns in the thermal limits of respiration (*T*_max_) and maximum respiration rates (*R*_max_). Respiration increases with rising temperature until it reaches a maximum (*R*_max_), and beyond this, respiratory function declines quickly (O’Sullivan et al. 2013; Scafaro et al. 2021). Across 218 species, *T*_max_ increased from 51 °C in the cold, high latitudes of the Arctic to 60.6 °C in hot, tropical rainforests (O’Sullivan et al. 2017). This relationship between *T*_max_ and temperature has been confirmed using a variety of growth temperature metrics (e.g. Zhu et al. 2021); however, the increase in *T*_max_ is smaller than the increase in temperatures from the Arctic to the tropics (O’Sullivan et al. 2017). To explore this mismatch, O’Sullivan et al. (2017) calculated thermal safety margins (the difference between *T*_max_ and heat-wave temperatures) and showed that mid-latitude sites with high heatwave temperatures have the narrowest thermal safety margins. Consequently, these sites are most likely to experience leaf damage from heatwaves both now and in the future as heatwaves become hotter and more frequent.

While biogeographic patterns in *T*_max_ have been explored, variation in *R*_max_ is still poorly understood. The *R*_max_ represents the biological ceiling for respiration in a given plant, and provides a single metric that encompasses respiratory enzyme concentrations, the temperature sensitivity of the respiratory components, the ability to supply substrates to the respiratory machinery at high temperatures, and demand for respiratory products. However, it is not yet known whether leaf *R*_max_ varies systematically across plants around the globe. *R*_max_ appears to be impacted by factors including drought, temperature, and fertilization (Gauthier et al. 2014; Heskel et al. 2014b), and to correspond to proxies for growth temperature, such as latitude (Griffin et al. 2022). Here we reanalyze the data from O’Sullivan et al. (2017) to extract *R*_max_ instead of *T*_max_, generating an *R*_max_ data set from 202 species spanning 19 sites across the globe (207 unique species-site combinations). Data were combined with site-level environmental data from O’Sullivan et al. (2017) including latitude (as a commonly used proxy for temperature), biome, mean maximum of the daily air temperatures of the warmest month (MMTWM), and mean annual precipitation, and leaf traits including LMA and leaf [N] and phosphorus concentrations ([P]) (see O’Sullivan et al. (2017) for methods). We analyzed relationships between site-mean data of mass- and area- based *R*_max_ (*R*_max-mass_ and *R*_max-area_; respectively) and environmental parameters using linear regression. We also examined relationships between *R*_max_ and mass- and area-based respiration at 25 °C (*R*_25-mass_ and *R*_25-area_, respectively) and leaf traits by performing standardized major axis regression on site-species mean data (note that 1 site, Cape Tribulation, QLD, was removed due to outlier in leaf [N] and [P] for all leaf trait analyses). All analyses took place in R v. 4.1.3 (R Core Team 2022). Standardized major axis regression analysis used the smatr package in R (Warton et al. 2012).

**Figure 2.**
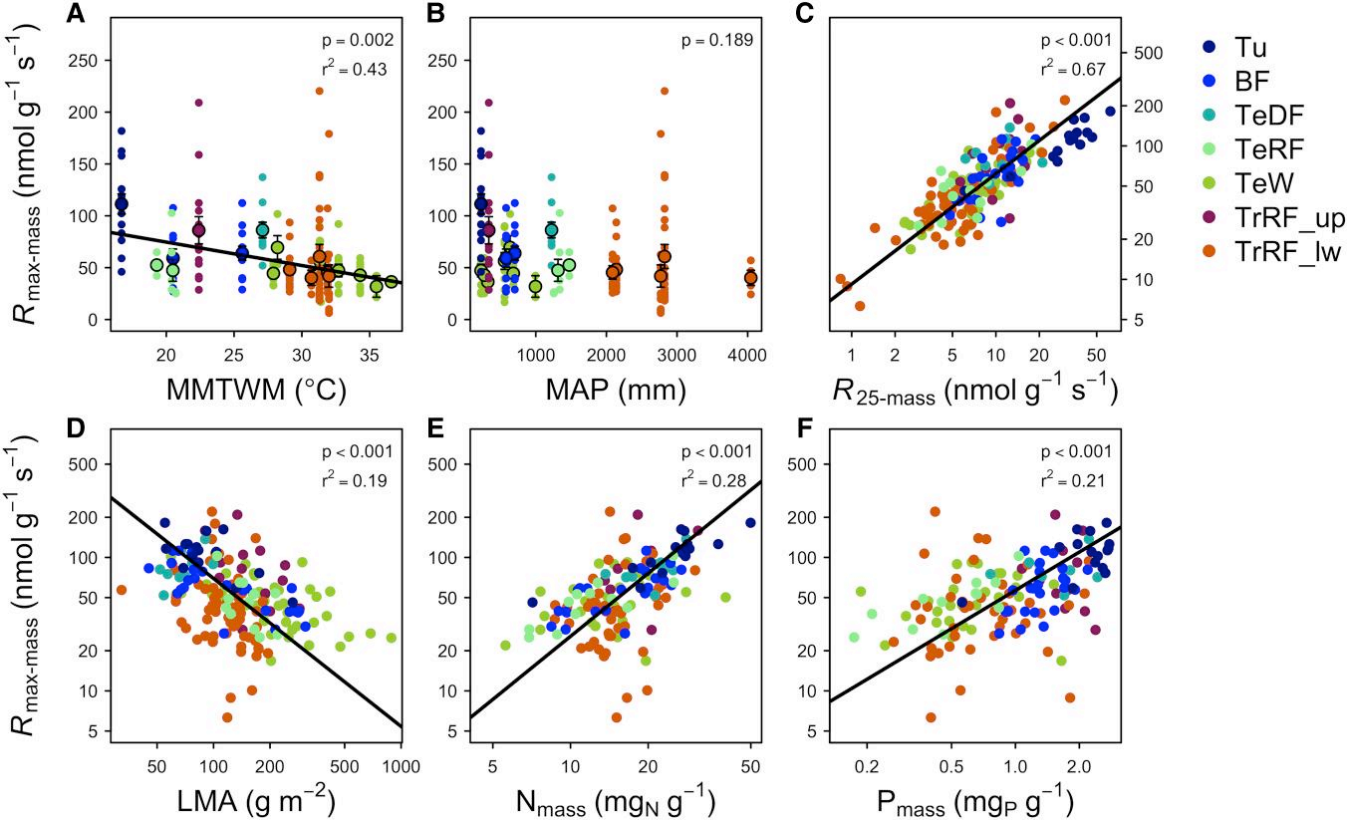
Relationships between mass-based maximum respiration rates (*R*_max-mass_) of plants and environmental variables, basal respiration, and leaf traits. These include **A)** mean maximum temperature of the warmest month (MMTWM), **B)** mean annual precipitation (MAP), **C)** mass-based respiration at 25 °C (*R*_25-mass_), **D)** leaf mass per area (LMA), **E)** mass-based leaf nitrogen (N_mass_), and **F)** mass-based phosphorus (P_mass_). The linear regression in **A)** (*P* < 0.05; see Table 1 for the equation) is through site-mean data (larger points; means ± SE, *n* = 19). Smaller points indicate species-site mean data (*n* = 191 for **A)**, **B)**, and **D)**; *n* = 190 for **C);** and *n* = 142 for **E)** and **F)**). In **C)**–**F)**, standardized major axis regressions through site-species mean data show significant relationships (*P* < 0.05; see Table 2 for equations). Abbreviations for biomes are as follows: Tu, tundra; BF, boreal forest; TeDF, temperate deciduous forest; TeRF, temperate rainforest; TeW, temperate woodland; TrRF_up, high elevation tropical rainforest; TrRF_lw, lowland tropical rainforest.

The *R*_max-mass_ had a negative relationship with MMTWM and a positive relationship with latitude (Fig. 2 and Supplemental Fig. S1; Table 1), such that the highest *R*_max_ was seen in cold, high latitude regions. Values of *R*_max-area_ also had a negative relationship with mean annual precipitation (Supplemental Fig. S2; Table 1). *R*_max_ is positively correlated to *R*_25_ on a mass- and area-basis (Fig. 2 and Supplemental Fig. S2; Table 2) and follows expected patterns in the leaf economic spectrum (Wright et al. 2004): LMA is negatively related with *R*_max-mass_, but positively related with *R*_max-area_, and we found positive relationships with *R*_max_ and leaf [N] and [P], both on a mass- and area-basis (Fig. 2 and Supplemental Fig. S2; Table 2). Our analysis indicates that *R*_25_ is a strong predictor of *R*_max_, but that there is still considerable spread in the relationship, due to variation in *T*_max_ and heat tolerance across species. Furthermore, *R*_max_ conforms to patterns previously observed in leaf basal respiration (Atkin et al. 2015), suggesting consistent patterns of variation to environmental gradients. However, of the environmental characteristics examined, temperature was best correlated with basal respiration (*R*_25_; Atkin et al. 2015), while mean annual precipitation explains more of the variance in *R*_max_ than does MMTWM. Together these results imply that the maximum metabolic capacity for respiration and leaf respiration rates under moderate conditions may be predominantly controlled by different environmental factors.

**Table 1.** Equations of the linear relationships (*y = mx + b*) shown in Fig. 2, Supplemental Fig. S1, and Supplemental Fig. S2

*y*	*x*	*m*	*b*
*R*_max-mass_	Absolute latitude	0.6429	34.1049
*R*_max-mass_	Mean max *T* warmest month	−2.2619	119.9493
*R*_max-area_	Mean annual precipitation	−0.0023	10.9375

**Table 2.** Equations of the standardized major axis regressions (*y = mx + b*) shown in Fig. 2 and Supplemental Fig. S2

*y*	*x*	*m*	*b*
*R*_max-mass_	*R*_25-mass_	0.8273	0.9644
*R*_max-mass_	LMA	−1.109	4.0603
*R*_max-mass_	N_mass_	1.5698	−0.161
*R*_max-mass_	P_mass_	0.9533	1.7535
*R*_max-area_	*R*_25-area_	0.9096	0.8198
*R*_max-area_	LMA	1.1082	−1.5273
*R*_max-area_	N_area_	1.5458	0.3462
*R*_max-area_	P_area_	0.9533	1.7535

## Patterns of respiration across biomes and PFTs

Efforts to understand global patterns of respiration have mainly focused on leaves. Leaf respiration per unit leaf area is often greater at cold sites when measured at a common temperature (Stocker 1935; Wager 1941; Van De Weg et al. 2012; Xiang et al. 2013; Atkin et al. 2015; Griffin et al. 2022). However, mass-based respiration measured at 25 °C can be similar in cold and warm sites (Reich et al. 1998; Wright et al. 2006), emphasizing the role of changes in LMA and structural tissue when interpreting respiration data. Global patterns of respiration can also be examined at the plant's growth temperature. In contrast to the increase in leaf respiration with increasing latitude found for basal respiration, respiration measured at the temperature of the warmest quarter was highest in the hot tropical and temperate mid-latitude sites (Atkin et al. 2015).

Leaf-level studies have provided important information on how respiration varies across PFTs, which group plants by characteristics such as growth form or leaf lifespan (Fisher et al. 2014), and are widely employed in terrestrial biosphere models. Leaf respiration varies with leaf lifespan and LMA (Wright et al. 2004), growth form (Slot et al. 2014), and leaf morphology (Schmiege et al. 2021). PFT-based variation in respiration was observed in a continental-scale study of leaf carbon cycling (Smith and Dukes 2018), and in a global-scale analysis of leaf respiration (Atkin et al. 2015). For example, basal respiration was higher in C_3_ herbs/grasses than in needle-leaved trees, broad-leaved trees, or shrubs. Similarly, for any given leaf [N], C_3_ herbs/grasses had higher leaf respiration rates than other PFTs (Atkin et al. 2015; but see Reich et al. 2008).

In contrast to these data for C_3_ species, we lack information on how respiration varies in C_4_ vegetation. Many terrestrial biosphere models parameterize leaf respiration in C_4_s based on a relationship between respiration, leaf [N], and the maximum carboxylation rate of Rubisco (Penning de Vries 1975; Bouma et al. 1995; Amthor 2000; Atkin et al. 2017) derived from maize (*Zea mays*) grown under optimal lab conditions (Collatz et al. 1992). Thus, we lack information on how respiration varies within C_4_ functional types (e.g. C_4_ grasses vs. C_4_ eudicots) and native C_4_ species in the field. Furthermore, maize is a member of the C_4_ NADP-dependent malic enzyme (NADP-ME) subtype and may not be representative of other C_4_ subtypes (Fan et al. 2022b). The C_4_ NAD-dependent malic enzyme (NAD-ME) and PEP-carboxykinase (PCK) subtypes involve mitochondria in their C_4_ cycle, affecting demand for respiratory products and, possibly, respiration rate (Fan et al. 2022a). Although no systematic differences in respiration among these C_4_ subtypes have been found, respiration did respond differently to growth temperature between the C_4_ subtypes (Fan et al. 2022a).

Variation in root respiration across biomes and PFTs has received less attention than leaf respiration. Field studies show that tree root respiration follows a similar pattern to leaves, with higher basal respiration rates in cold, high latitudes than in warm, low latitudes (Burton et al. 2002; Burton et al. 2008). There is also a burgeoning understanding of how root respiration varies across PFTs. A recent study examining respiration of fine roots at 20 °C in 245 species found no differences in respiration between woody and nonwoody species. However, within woody species, deciduous species had higher root respiration rates than evergreens (Han and Zhu 2021). Overall, root respiration varies greatly across species, but differences across PFTs have been hard to identify, likely due to the challenges of measuring root respiration, as well as variability in root size, length and other morphological characteristics, and growth environments (including soil temperature and mycorrhizal associations) (see Box 3).

Box 3. **Global patterns in root respiration**

**Figure 3.**
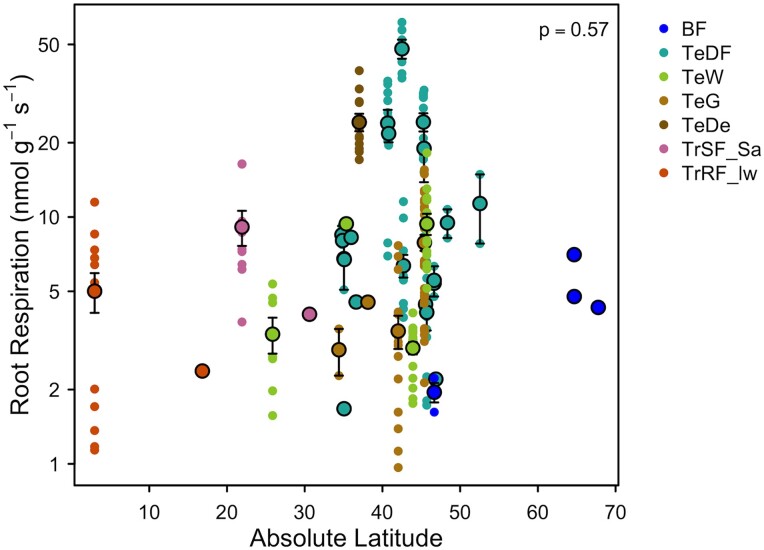
Root respiration rates as a function of latitude. No significant relationship was found through site-mean data (larger points, means ± SE, *n* = 37). Smaller points indicate species-site mean data (*n* = 206). Abbreviations for the biomes are as follows: BF, boreal forest; TeDF, temperate deciduous forest; TeW, temperate woodland; TeG, temperate grassland; TeDe, Temperate desert; TrSF_Sa, Tropical seasonal forest/savanna; TrRF_lw, lowland tropical rainforest.

Han and Zhu (2021) compiled one of the largest databases of root respiration to date. Here, we use their data to examine the hypothesis that root respiration will follow established patterns of leaf respiration, with higher respiration (measured at a common temperature of 20 °C) at higher latitude, colder sites. We narrowed the Han and Zhu (2021) data set to studies carried out in fields, removing data from plants grown in pots. We then assigned biomes to each species based on the study location and calculated the mean root respiration rate for each species (i.e. species mean) and at each site (i.e. site mean). We used the site-mean data and linear regression [performed in R v. 4.1.3 (R Core Team 2022)] to assess relationships between latitude (as a commonly measured proxy for temperature, available from the original data set) and root respiration at 20 °C. Contrary to our hypothesis, we found no significant relationship between root respiration measured at 20 °C and latitude (Fig. 3; *P* > 0.05). It is possible that as more data on root respiration become available, such a relationship may be uncovered. However, we suspect that latitudinal patterns in root respiration will be more difficult to identify than in leaves. For example, we know that latitude and air temperature co-vary, which explains the latitudinal patterns found in leaf respiration rates. Yet, soil temperatures are frequently offset from air temperatures, and the extent of this offset is also impacted by soil moisture (Lembrechts et al. 2022), which may confound the types of global patterns we see in root respiration.

## Drought sensitivity of respiration

The impact of drought on plant function is increasingly of concern due to the increasing frequency and severity of drought events (Spinoni et al. 2014). Water stress decreases both root (Burton et al. 1998; Bryla et al. 2001) and whole-plant respiration (De Vries et al. 1979). In contrast, the effect of drought on respiration of mature leaves is variable. Studies show increases (Metcalfe et al. 2010; Rowland et al. 2015), no change (Gimeno et al. 2010), and decreases (Rodríguez-Calcerrada et al. 2010; Ayub et al. 2011; Crous et al. 2011; Sevanto et al. 2014; Collins et al. 2021) in foliar respiration during drought (Atkin and Macherel 2009), although drought-induced reductions in respiration are common. Variation in leaf respiratory drought responses may be caused by differences between species, or interactions with other environmental variables (Flexas et al. 2005). A test of the first two possibilities showed that herbs and short-lived species show a greater decline in respiration with drought than species with longer-lived leaves (Galmés et al. 2007). Alternatively, variation in respiratory drought responses may be explained by differences in drought severity and length. Flexas et al. (2005) hypothesized that respiration responses to drought are biphasic, with an initial reduction in respiration due to reductions in growth respiration and a subsequent increase in respiration due to increased maintenance respiration. Initial drought-induced decreases in respiration are likely driven by reduced substrate supply (because photosynthesis is downregulated more than growth and respiration) or reduced demand for respiratory products (Atkin and Macherel 2009), though since sugar concentrations usually do not change with drought, a demand limitation is the more likely driver (Ayub et al. 2011; Crous et al. 2011). Later increases in respiration may be caused by the need for increased ATP to fuel protein maintenance during prolonged drought (Slot et al. 2008; Rowland et al. 2015).

Importantly, the majority of data on respiratory responses to drought come from potted plants (Bartoli et al. 2005; Galmés et al. 2007; Ayub et al. 2011) or manipulative through-fall experiments (Rowland et al. 2015; Collins et al. 2021). These studies provide data on respiratory responses across a variety of species, with a focus on regions experiencing or projected to experience future drought (Amazon basin, Metcalfe et al. 2010; Doughty et al. 2015; Rowland et al. 2015; Mediterranean species, Galmés et al. 2007; southwestern United States, Sevanto et al. 2014; Collins et al. 2021). Interestingly, results from naturally occurring vegetation are generally opposite to those from drought manipulation experiments, where artificial rooting environments (primarily in pot studies) can introduce challenges for extrapolating to natural systems. Instead, natural studies and cross-site comparisons show increases in leaf respiration as water availability decreases (Turnbull et al. 2001; Wright et al. 2006; Atkin et al. 2015). Our data on *R*_max_ from natural vegetation concur with these results. Overall, the implication is that timescales matter: long-term acclimation or evolution to aridity is not the same, physiologically, as a rapidly imposed drought from a well-watered baseline.

## Respiration response to elevated CO_2_ concentrations

Respiratory responses to rising atmospheric CO_2_ concentrations ([CO_2_]) are highly variable (Smith 2017; Dusenge et al. 2019). While short-term fluctuations in [CO_2_] do not affect leaf respiration (Amthor 2000), long-term exposure to high [CO_2_] can stimulate (Wang et al. 2001; Crous et al. 2011), suppress (Curtis 1996; Noguchi et al. 2018), or have weak or no effect on leaf respiration rates (Tjoelker et al. 2001a; Gauthier et al. 2014; Lauriks et al. 2021), and no effect on stem respiration rates (Mildner et al. 2015; Salomón et al. 2019). Indeed, even within a single study, the response of respiration to elevated [CO_2_] can vary between species (Hamilton et al. 2001; Sanhueza et al. 2022). Multiple hypotheses have been put forward for these results, including reduced leaf respiratory demand due to lower Rubisco concentrations (Ainsworth and Long 2005), and stimulation of respiration due to enhanced carbohydrate availability (Rogers et al. 2004) or upregulation of respiratory gene transcription (Li et al. 2013; Markelz et al. 2014). No one hypothesis to date can explain all the data. Future work in this area is clearly needed to improve our understanding of how rising [CO_2_] will alter respiration in vegetation.

## Conclusions

Ecophysiological studies on plants in intact ecosystems have provided numerous insights on respiration. From these, emergent properties and environmental controls on respiratory fluxes have been characterized, allowing us to understand this important process and ecosystem carbon flux. However, much remains unknown about how respiration varies within and across plant species—especially in environments that are not experimentally controlled (see “Outstanding Questions”). As we scale up from plant tissue processes to ecosystem scale fluxes, and from instantaneous responses to annual responses, the dominant drivers of respiration shift from those associated with short-term tissue physiology to those that constrain substrate provision on annual (and longer) time scales. While we have yet to develop the ability to pull together these various strands of respiration across spatial and temporal scales, understanding how respiration responds to its environment across biological scales will only become more important as we move into a warmer, drier, high CO_2_ world.

The majority of the studies surveyed for this review prioritize measurements of healthy, sun-lit leaves. While this allows for direct comparisons, it also restricts the ability to capture heterogeneity in the biology and environment of many organisms. The neglect of measurements of shade leaves or leaves experiencing disturbances presents respiration in a semi-idealized state, and may lead to inaccurate estimations of ecosystem carbon fluxes (Keenan and Niinemets 2016; He et al. 2018).

Similarly, we know much more about respiration in leaves than in stems or roots. However, to better model and predict respiration in vegetation will require us to expand our knowledge of how CO_2_ effluxes in these plant tissues differ from those of leaves. More efforts towards developing broad-scale, global patterns in stem and root respiration are therefore needed, including data sets from the field to support these syntheses.

Ecophysiologists and modelers can benefit greatly from respiration data sets that span plants representing diverse ecologies, climates, evolutionary histories, and functional groups (Atkin et al. 2015; Heskel et al. 2016; O’Sullivan et al. 2017; Smith and Dukes 2018). We acknowledge the limitations within these data sets though, given the logistical and financial limitations of field work—not all “global” data are evenly sourced throughout the globe. However, learning from the emergent properties of these data allows for improved sampling in the future, as well as expanding ecophysiological surveys to include disturbances and environmental variability. Additionally, we advocate for a move towards relating global patterns in respiration with environmental variables that directly affect plant performance (such as temperatures or precipitation during the growing season) rather than commonly used, but biological less relevant variables such as mean annual temperature, mean annual precipitation, and latitude. Collectively, a more refined and mechanistic understanding of plant respiration for global processes depends on a combination of field studies on intact and altered ecosystems, as well as the integration of knowledge from controlled studies and managed species.

**Advances**

- Recently developed methods for collecting respiration aim to increase measurement efficiency (using a high-throughput fluorometric oxygen sensor) and spatial coverage (using hyperspectral data).
- Maximum leaf metabolic capacity correlates well with the more commonly measured respiration rates at 25 °C, environmental predictors, including temperature and precipitation, and leaf traits, including nitrogen, phosphorus, and leaf mass per area.
- Recent root respiration data compilations lay the groundwork to expand beyond our current leaf-centric understanding of global patterns of respiration across biomes, latitudes, and PFTs.
- Recent advances in plant respiration modeling include incorporating diel variation into temperature functions used to model nocturnal plant respiration, modifications in up-scaling local to stand-level measurements, and improved modeling of stem respiration.

**Outstanding questions**

- How well do respiration measurements from healthy, mature sun leaves represent the broader range of leaves found in natural systems?
- Do stem and root respiration rates follow global patterns similar to leaf respiration?
- How do respiration rates vary among PFTs (e.g. C_3_ vs. C_4_ plants), and how can this variation be parameterized in models?
- Can we improve our ability to estimate respiration across broad spatial scales in natural systems by developing new remote sensing techniques?
- How will climate change, including warming, rising CO_2_ concentrations, and drought, impact respiration in natural ecosystems?

## Supplementary Material

kiad167_Supplementary_Data

## Data Availability

The data set on *R*_max_ will be made publicly available through Dryad Digital Repositories on acceptance of the article.

## Supplemental data

The following materials are available in the online version of this article.

**Supplemental Figure S1**. Global pattern in mass-based maximum respiration rates (*R*_max-mass_) of plants versus absolute latitude.

**Supplemental Figure S2**. Relationships between area-based maximum respiration rates (*R*_max-area_) of plants and environmental variables, basal respiration and leaf traits.
