# Supplementary material for: It's only natural: Plant respiration in unmanaged systems
Source: Plant Physiol. 2023 Mar 21;192(2):710–27. doi: 10.1093/plphys/kiad167 (PMC10231469; doi:10.1093/plphys/kiad167)
Supplement: kiad167_Supplementary_Data [file kiad167_supplementary_data.pdf]

SUPPLEMENTAL INFORMATION

It's only natural: plant respiration in unmanaged systems

Stephanie C. Schmiege[1,2], Mary Heskel[3], Yuzhen Fan[4], Danielle A. Way[2,4,5,6]*

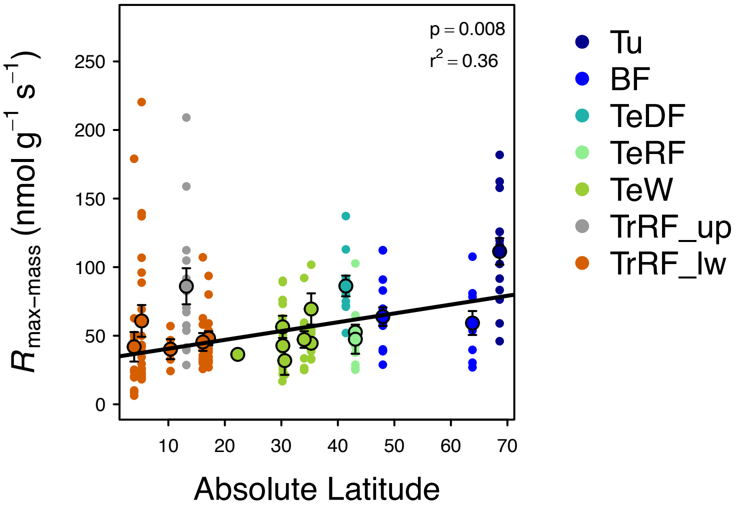

Supplemental Figure S1. Global pattern in mass-based maximum respiration rates ($R_{max-mass}$) of plants versus absolute latitude. The linear regression is through the site-mean data (larger points, means ± SE; $p<0.05$, n = 19). See Table 1 for equations. Smaller points indicate species-site mean data (n = 191). The high elevation tropical rainforest site in Peru was excluded from analyses (greyed-out points) following O'Sullivan et al. (2017), as it is a high altitude site. Abbreviations for biomes are as follows: Tu, tundra; BF, boreal forest; TeDF, temperate deciduous forest; TeRF, temperate rainforest; TeW, temperate woodland; TrRF_up, high elevation tropical rainforest; TrRF_lw, lowland tropical rainforest.

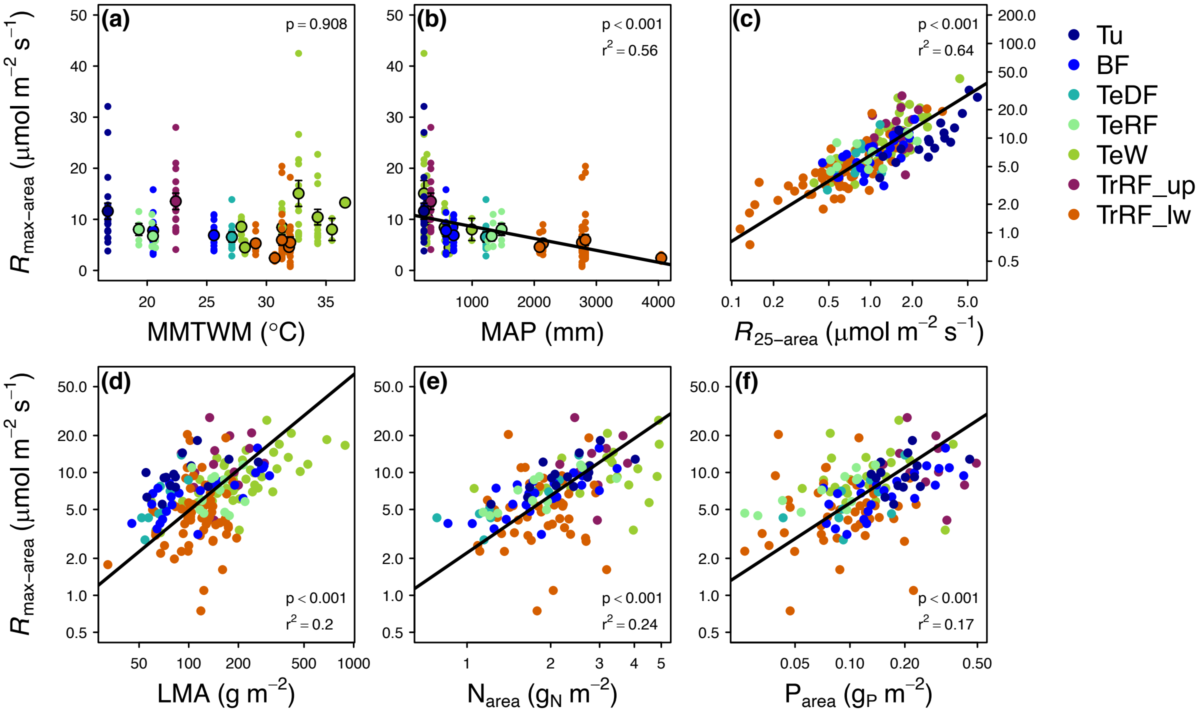

Supplemental Figure S2. Relationships between area-based maximum respiration rates ($R_{max\text{-}area}$) of plants and environmental variables, basal respiration and leaf traits. These include: (a) mean maximum temperature of the warmest month (MMTWM), (b) mean annual precipitation (MAP), (c) area-based respiration at 25 °C ($R_{25\text{-}area}$), (d) leaf mass per area (LMA), (e) area-based leaf nitrogen (N_{area}), and (f) area-based phosphorus (P_{area}). Linear regressions in (a) and (b) are through site-mean data (larger points, means ± SE, n = 19) with regression lines only shown for significant relationships ($p < 0.05$; see Table 1 for equations). Smaller points indicate species-site mean data (n = 207 for (a, b), n = 206 for (c), n = 191 for (d) and n = 142 for (e, f)). In (c-f), standardized major axis regressions through site-species mean data show significant relationships ($p < 0.05$; see Table 2 for equations). Abbreviations for biomes are as follows: Tu, tundra; BF, boreal forest; TeDF, temperate deciduous forest; TeRF, temperate rainforest; TeW, temperate woodland; TrRF_up, high elevation tropical rainforest; TrRF_lw, lowland tropical rainforest.
